# Monitoring changes of docosahexaenoic acid-containing lipids during the recovery process of traumatic brain injury in rat using mass spectrometry imaging

**DOI:** 10.1038/s41598-017-05446-2

**Published:** 2017-07-11

**Authors:** Shuai Guo, Dan Zhou, Mo Zhang, Tiejun Li, Yujie Liu, Yupin Xu, Tianjing Chen, Zhili Li

**Affiliations:** 1 Department of Biophysics and Structural Biology, Institute of Basic Medical Sciences, Chinese Academy of Medical Sciences & School of Basic Medicine, Peking Union Medical College, Beijing, P.R. China; 2 School of Pharmacy, Second Military Medical University, Shanghai, P.R. China

## Abstract

Brain lipid homoeostasis is critical during recovery process after traumatic brain injury (TBI). In this study, we integrated liquid extraction and electrosonic spray ionization technology to develop an ionization device coupled with a Fourier transform ion cyclotron resonance mass spectrometer for imaging of docosahexaenoic acid (DHA)-containing lipids on rat brain tissues. The ion images of the brain tissue sections from the normal rats and the rats after TBI at acute phase (0 and 1 day) and chronic phase (3, 5, and 7 days) were obtained. The imaging results indicate that the levels of DHA and lyso-phosphatidylethanolamine (22:6) in the injury area of TBI rats increased significantly at the acute phase and subsequently decreased at the chronic phase. But the levels of DHA-containing phospholipids including phosphatidylethanolamine (PE)(P-18:0/22:6), PE(18:0/22:6), and phosphatidylserine (18:0/22:6) decreased at the acute phase and gradually increased at the chronic phase in the injury area accompanied by the morphogenesis and wound healing. These findings indicate that the DHA may participate in the recovery process of brain injury. This is the first report to *in situ* detect the changes in the levels of DHA and DHA-containing lipids in the TBI model.

## Introduction

Traumatic brain injury (TBI) is a major cause of death and permanent disability for people under the age of 45^1–3^. This injury occurs frequently in military personnel and professional athletes, leading to loss of limb function, speech impairment, memory disturbances, and emotional responses^4^. It is a multifaceted disease with prolonged secondary pathogenesis of excitotoxicity, oxidative stress, inflammation, and long-lasting adverse neurological sequelae such as secondary epilepsy, chronic headaches, post-traumatic stress disorder, neurocognitive deficit, as well as neurodegenerative diseases of Alzheimer’s disease or Parkinsonism^5–7^. Current TBI treatments focus on the management of intracranial pressure, the prevention and treatment of hypotension, and adequate ventilation, but no specific medical treatment is provided specifically for neuroprotection and recovery^8^.

Omega-3 fatty acids are essential polyunsaturated fatty acids and they can not be synthesized by human body^9^. Docosahexaenoic acid (FA(22:6), DHA) as a major omega-3 fatty acid in neural tissue has an important role in the structure and function of the brain^10^. Accumulating evidences suggest that DHA, highly enriched in fish oil^11^, has therapeutic potential in neurotrauma^12–14^. Recent animal studies have demonstrated that dietary or intravenous supplementation with DHA either before or after TBI improves functional outcomes^15–18^. Mechanistic investigations suggest that DHA influences multiple aspects of the pathologic molecular signaling cascade including decreased neuroinflammation and oxidative stress, neurotrophic support, and the activation of cell survival pathways^13, 14^. Increased plasma level of DHA has been observed at day 1 but decreased 3 days after injury^19^. However, histological evidence of its distribution on TBI tissue is still lacking.

Mass spectrometry imaging (MSI) is a powerful tool to directly observe the distributions of various molecules, such as proteins, peptides, lipids, metabolites, and drugs on complex biological matrixes^20^. MSI can image chemical species without prior knowledge, indicating its potential to *in situ* investigate multiple biomolecules related to cancers, neurological diseases, and various biological phenomena on tissues^21–24^. Currently, matrix-assisted laser desorption/ionization (MALDI) is a major technique for MSI due to its good salt tolerance, high spatial resolution, and wide mass range^25–27^. When equipped with a high mass resolution mass spectrometer such as Fourier transform ion cyclotron resonance mass spectrometer(FTICR MS), ion images of multiple peaks with high mass accuracy can be obtained^28, 29^. However, this method has the challenges of complex sample preparation prior to analysis and the interferences of matrixes and/or matrix adducts for the detection of small molecules such as DHA.

To overcome the drawbacks of MALDI for MSI, ambient mass spectrometry is undergoing rapid development because it can detect chemical species with minimal sample preparation prior to analysis and has no background interferences in low mass region^30, 31^. Most commonly used ambient ionization technique such as desorption electrospray ionization (DESI) can ionize chemical species such as metabolites, lipids, and peptides on sample surfaces with a spatial resolution of approximately 180 μm^32^. Recently, several liquid extraction techniques have been developed for simple operation and fast analysis^33^. Among them, liquid microjunction surface sampling probe is performed via the extraction and ionization process using a concentric dual-capillary applied for analyzing chemical species dissolved in probe-to-surface liquid junction with a spatial resolution of approximately 650 μm^34^. NanoDESI with a smaller liquid junction of 8 μm has overcome the spatial resolution limitation of liquid extraction-based approach. This technique employs two angled capillaries to form a liquid bridge on a sample surface, achieving a lateral resolution of 12 μm or even single cell level analysis^35–37^.

Electrosonic spray ionization (ESSI) was developed by a combination of a traditional electrospray ionization source and supersonic nebulizing gas^38^. Previous studies suggest that ESSI has a comparable sensitivity with nanospray and a great flexibility for the detection of multiple species, especially for enzyme-substrate complexes^39, 40^. In this study, we combined ESSI source with the liquid bridge to form a liquid extraction-ESSI (LE-ESSI) device for ambient MSI. Our experimental results indicate that the LE-ESSI has the ability for *in situ* analysis of lipids in complex biological matrixes, along with high reproducibility and good linear correlation. Due to less background interference in low mass region, this device coupled with FTICR MS was used to investigate changes in the levels of DHA and DHA-containing lipids, such as lyso-phosphatidylethanolamine (LPE)(22:6), lyso-phosphoglycerols (LPG)(22:6), phosphatidylethanolamine (PE)(P-18:0/22:6), PE(18:0/22:6), and phosphatidylserines (PS)(18:0/22:6), in the brain tissue sections of normal rats and the rats after TBI at 0, 1, 3, 5, and 7 days post-injury.

## Results

### Optimizing parameters of the LE-ESSI

**Figure 1:**
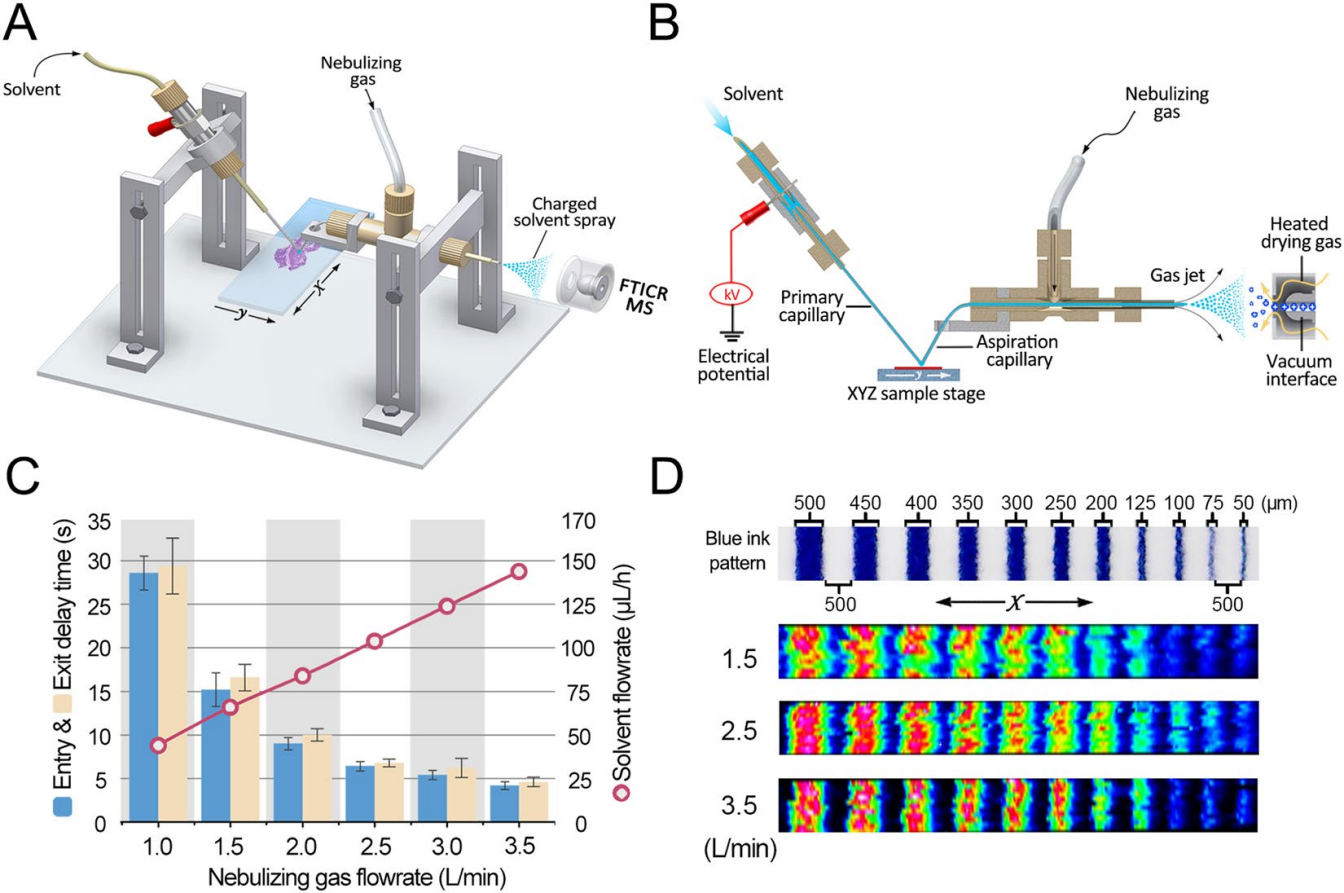
Experimental setup and the optimization of nebulizing gas flowrate and extracted solvent flowrate of the LE-ESSI. (**A**) 3D view schematic representation of the LE-ESSI device. (**B**) Schematic diagram of LE-ESSI MSI. (**C**) The entry & exit delay time of the extracted analytes when liquid bridge was located into or out off of the blue ink band at different nebulizing gas flowrate and different solvent flowrate. (**D**) Optical images and ion images of the blue ink patterns at different nebulizing gas flowrate.

The schematic diagram and physical photo of the LE-ESSI are shown in Fig. 1A,B and Supplementary Figure S1, respectively. Previous study suggested that the droplet size formed by liquid bridge and the hysteresis for imaging are dependent on the flowrate of extraction solution^36^. In our study, we observed that the nebulizing gas flowrate was closely correlated with the solvent flowrate because of the Venturi self-pumping effect. As shown in Fig. 1C,D, the nebulizing gas flowrate and the solvent flowrate were optimized in positive ion mode based on the blue ink bands. It was found that the nebulizing gas flowrate of 3.5 L/min and the solvent flowrate of 145 μL/h could provide a short delay time (entry & exit) of the extracted analytes (Fig. 1C) and high quality ion image (*m/z* 645.5210) along the X-axis direction corresponding to the lateral spatial resolution of approximately 50 μm (Fig. 1D). The above-mentioned parameters were further evaluated using phosphatidylcholine (PC)(36:0) (3.2 fmol/μL, *m/z* 790.6320) as a compound model (Supplementary Figure S2) and homogeneous liver tissue section as a tissue section model (Supplementary Figure S3), respectively. It should be noted that the ion intensities were enhanced with an increase in the nebulizing gas flowrate and with a decrease in the distance between the spray tip and MS inlet. To get high quality ion images and to reduce the contamination of MS, the distance of 5 mm and the nebulizing gas flowrate of 3.5 L/min were adopted for the following analysis.

### Characteristics of the LE-ESSI imaging

**Figure 2:**
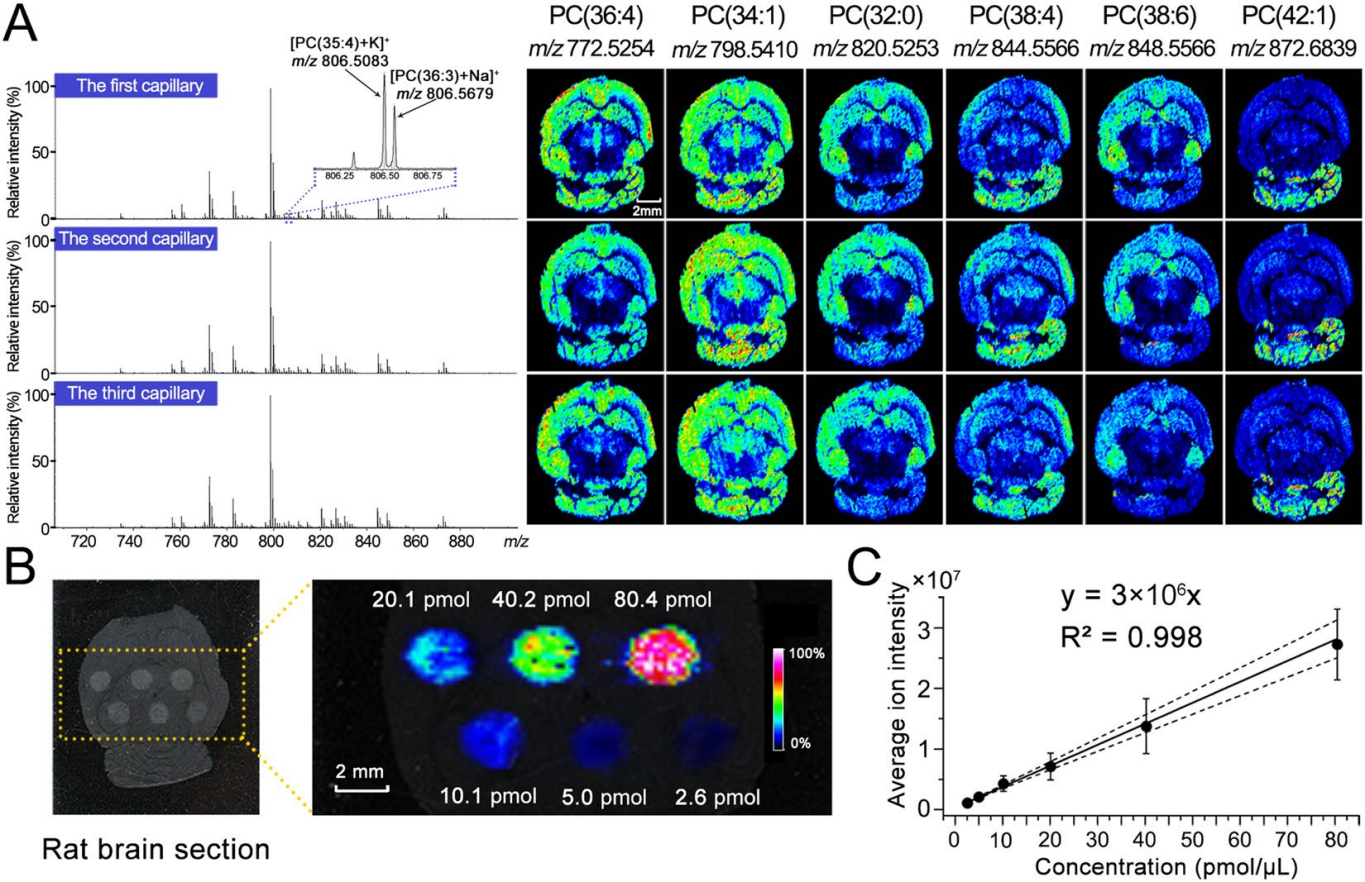
Tissue imaging of mouse brain sections and an exogenous lipid spotted on rat brain section in positive ion mode. (**A**) Average mass spectra and the corresponding ion images obtained from three adjacent tissue sections using three different aspiration capillaries, respectively, in a high mass resolution mode with a mass resolution of 200,000 (at *m/z* 400) and a spatial resolution of 150 μm. (**B**) Optical image and MSI result of different amounts (from 2.6 pmol to 80.4 pmol) of [PC(24:0)+K]^+^ (*m/z* 600.4001) spotted (1 μL) on a rat brain tissue section. (**C**) Standard curve by plotting the amounts of each spot against the average intensities of *m/z* 600.4001 each spot. Error bars represent the standard deviation of the intensity of *m/z* 600.4001 from about 130 mass spectra in each spot.

To assess the imaging capability of the LE-ESSI, three same aspiration capillaries (7 cm long) were subsequently employed to image chemical species on three adjacent brain tissue sections, respectively. As shown in Fig. 2A, the LE-ESSI operated in positive ion mode has exhibited a good reproducibility of ion images and their corresponding average mass spectra during three independent imaging experiments, along with a high mass resolution of 200,000 at *m/z* 400 and a spatial resolution of 150 μm. It should be pointed out that the LE-ESSI-FTICR MSI also allowed us to capture and distinguish two (or more) species with a slight mass difference ([PC(35:4)+K]^+^ at *m/z* 806.5083 and [PC(36:3)+Na]^+^ at *m/z* 806.5679).

To further assess the quantitative capability of the LE-ESSI on a complex biological matrix, as exemplified by an exogenous lipid, PC(24:0), its different amounts were manually spotted at different locations of a rat brain tissue section followed by performing LE-ESSI imaging at a lateral resolution of 150 μm. It was found that the enhanced intensity of [PC(24:0)+K]^+^ at *m/z* 600.4001 was observed with the increased amount of PC(24:0) on the brain tissue section (Fig. 2B). After averaging all mass spectra in each spot, it was found that the amounts of PC(24:0) on each spot linearly correlated with its corresponding ion intensities, along with a correlation coefficient of 0.998 (Fig. 2C). Taken together, our results indicate that the LE-ESSI has exhibited a potential ability to perform relative quantitative analysis of lipids in complex biological matrixes.

### Profiling DHA and DHA-containing lipids in normal rat brain tissues and the rat brain tissues after TBI

**Figure 3:**
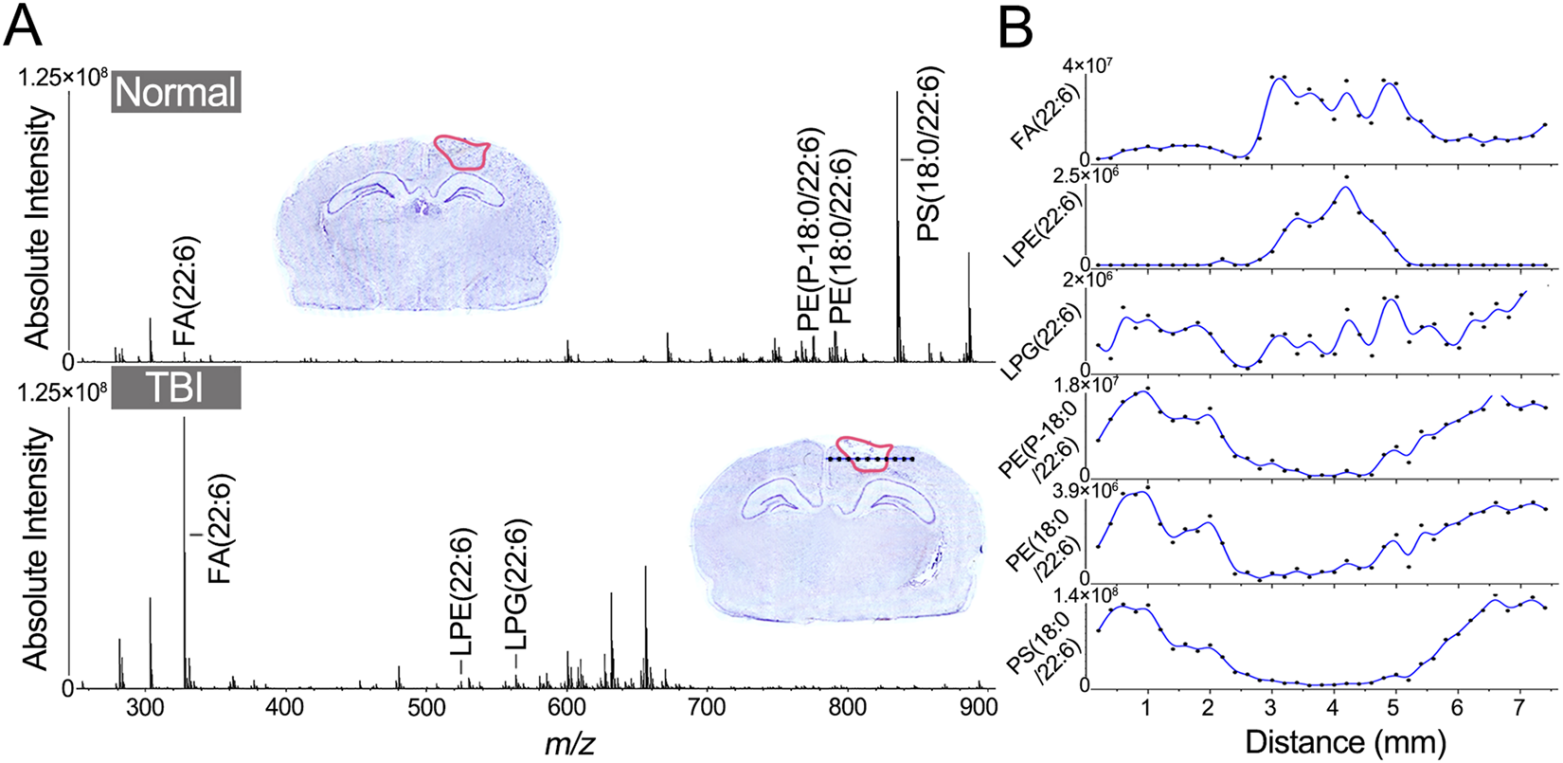
Tissue profiling on brain sections derived from TBI model and normal control. (**A**) Mass spectra acquired from the injury areas of TBI model (3 days post injury) and normal control. Cresyl violet staining of brain sections was shown in the side of mass spectra, and the injury area was circled with red line. (**B**) Plots of the absolute intensity of FA(22:6), LPE(22:6), LPG(22:6), PE(P-18:0/22:6), PE(18:0/22:6), and PS(18:0/22:6) along with the distance (mm) of black line which throughout the injury area of the optical image of TBI model of (**A**).

Lipids profiling was conducted in negative ion mode with a mass resolution of 66,000 at *m/z* 400, and a total of 94 lipids including fatty acids (FAs), diglycerides (DGs), PEs, phosphatidic acids (PAs), PSs, phosphoglycerols (PGs), and phosphatidylinositols (PIs) were identified on a rat brain tissue section (Supplementary Table S1). Among them, DHA and five DHA-containing lipid species including LPE(22:6), LPG(22:6), PE(P-18:0/22:6), PE(18:0/22:6), and PS(18:0/22:6) were reliably assigned based on their *in situ* tandem mass spectra on tissue section (Supplementary Figure S4). The fragment peek at *m/z* 327.2327 corresponds to FA(22:6) chain in the lipids species^41^. When imaging the injury area (circled with red line) of TBI model at 3 days post-injury and the corresponding area in normal control, typical negative ion spectra were acquired without any background interferences. As shown in Fig. 3A, representative mass spectra from the rat brain injury area and the corresponding brain area of normal control exhibit significant differences in the levels of FA(22:6), LPE(22:6), and LPG(22:6) over low mass range of 250~700 Da and in the levels of PE(P-18:0/22:6), PE(18:0/22:6), and PS(18:0/22:6) over the high mass range of 700~900 Da. To examine the correlation between the levels of the above-mentioned lipids and the degree of the injury, change trends in their absolute intensities were plotted against across the injury area (Fig. 3B). It was found that FA(22:6) and LPE(22:6) significantly increased and PE(P-18:0/22:6), PE(18:0/22:6), and PS(18:0/22:6) significantly decreased in the injury area compared with the adjacent normal areas.

### MSI of DHA and DHA-containing lipids in brain tissue sections of TBI rat and control rat

**Figure 4:**
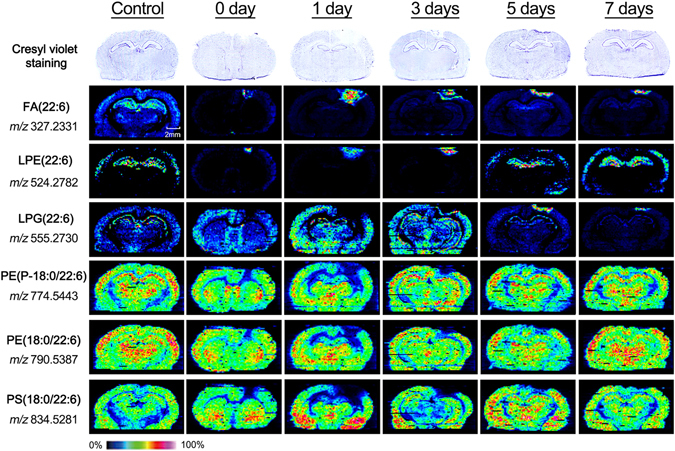
MSI of FA(22:6), LPE(22:6), LPG(22:6), PE(P-18:0/22:6), PE(18:0/22:6), and PS(18:0/22:6) in normal and TBI groups sacrificed at 0, 1, 3, 5, and 7 days after injury. Cresyl violet staining of brain sections was shown in above of the ion images.

MSI was performed on tissue sections derived from normal and TBI rats at the acute phase (0 and 1 day) and at chronic phase (3, 5, and 7 days). All these experiments were performed based on the brain tissue sections of three groups (Fig. 4 and Supplementary Figure S5). As shown in Fig. 4, FA(22:6) presents mainly in the injury areas at 1 and 3 days post-injury, and then its level gradually decreased during recovery. LPE(22:6) mainly located in the injury areas at 1 and 3 days, and increased LPE(22:6) appeared in the hippocampus in TBI samples at 5 and 7 days, which is similar to normal control. For LPG(22:6), its similar distributions at 0, 1, and 3 days on brain injury tissue sections and at normal brain tissue section were detected, while significantly increased LPG(22:6) was observed in the injury areas at 5 and 7 days. For PE(P-18:0/22:6), PE(18:0/22:6), and PS(18:0/22:6), their decreased levels at 0, 1, and 3 days and their increased levels at 5 and 7 days were detected in the injury area as compared with normal controls. In addition, if the distribution area of significantly increased FA(22:6) represents the injury area, it is found that the injury area at 1 day was significantly larger than that at 0, 3, 5,or 7 days post-injury (Supplementary Figure S6).

### Changes in the levels of DHA and DHA-containing lipid species during the recovery process of TBI rats

**Figure 5:**
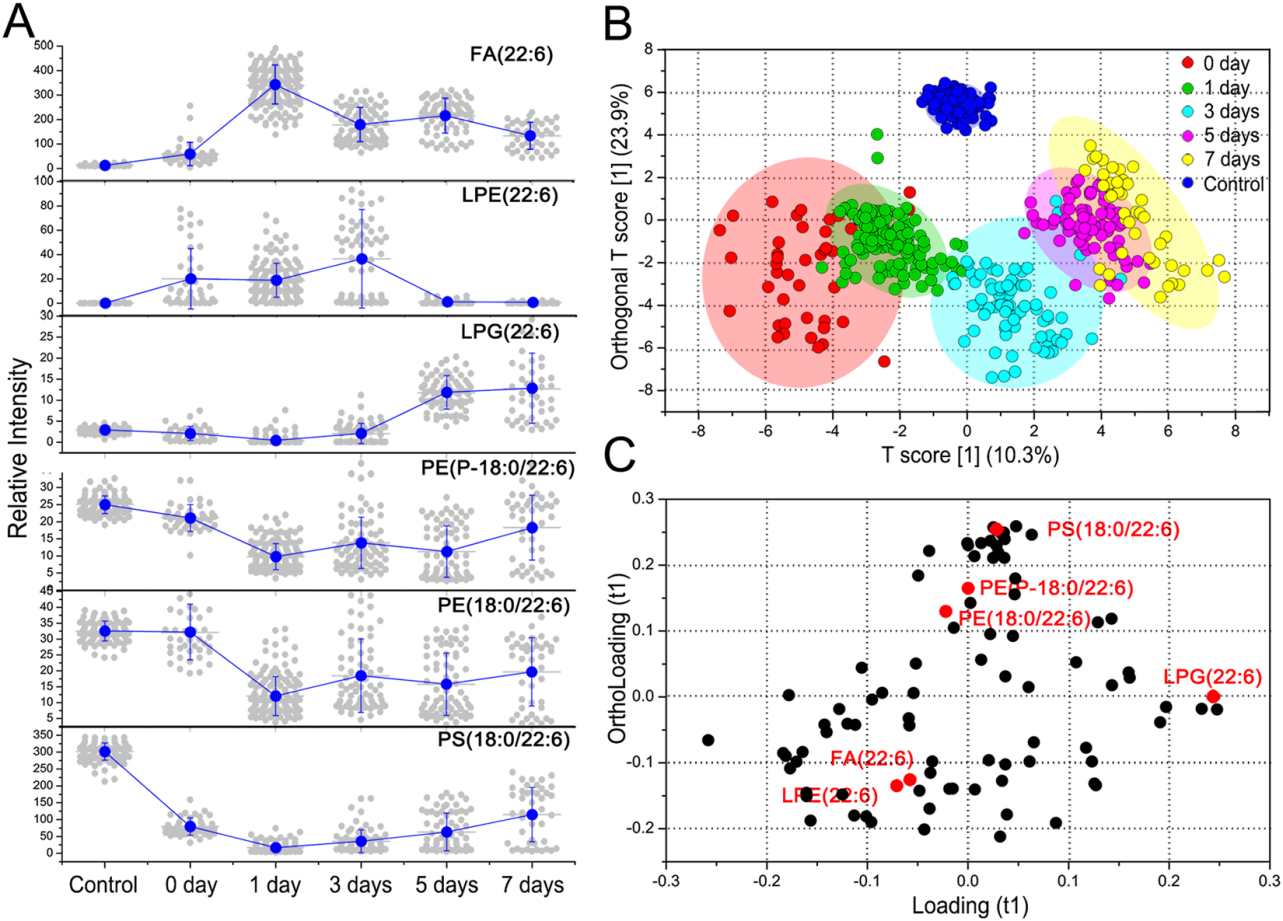
Statistic analysis for separation and feature selection of TBI groups with different time points and normal control. (**A**) Change tendencies of FA(22:6), LPE(22:6), PE(P-18:0/22:6), PE(18:0/22:6), and PS(18:0/22:6) in injury areas of TBI groups and normal control. (**B**) OPLS-DA score plots of lipids from the ROIs between normal control (blue) and TBI groups sacrificed at 0 day (red), 1 day (green), 3 day (light blue), 5 day (pink), and 7 day (yellow). (**C**) Loading plots of lipids from the TBI groups and normal control. DHA and DHA-containing lipid species were labeled in red.

The absolute intensities of 94 detected lipids in Fig. 4 were normalized against the total ion intensity of 1000 and the relative ion intensity of each lipid species in the injury area was calculated relative to the total ion intensity in each pixel. The sum of the ion intensities of all detected lipids in each pixel was defined as the level of the corresponding pixel and the sum of all pixel levels in each time point was termed as the level of the corresponding point. Changes in the levels of these lipids among the different time points, as well as normal control were analyzed using both univariate and multivariate statistics. As shown in Fig. 5A, the level of FA(22:6) significantly increased in the injury area at 1 day and then gradually decreased with the recovery of injury. LPE(22:6) and LPG(22:6) showed the opposite change trends in the injury area after 1 day. PE(P-18:0/22:6), PE(18:0/22:6), and PS(18:0/22:6) had lower level in injury areas at 1 and 3 days compared with the normal, and then gradual increase in their levels at 5 and 7 days was observed. These results of Fig. 5A are consistent with ion images of Fig. 4. In addition, normal control and TBI rats from different time points also remain statistically significant (Supplementary Table S2).

As shown in Fig. 5B, orthogonal partial least squares discriminant analysis (OPLS-DA) indicated that the injury rats at the different time points can be differentiated by the principal component (PC) 1, and the PC 2 could further differentiate the normal control from the injury rats. The loading plots show the significantly changed variables for distinguishing normal control from the injury tissues at the different time points (Fig. 5C **)**. LPG(22:6) located on the right side along the positive loading (t1) direction has a potential ability to differentiate the injury rats at the different time points. FA(22:6), LPE(22:6), PE(P-18:0/22:6), PE(18:0/22:6), and PG(18:0/22:6) located in the outside of ortholoading (t1) direction can distinguish the normal control from injury rats.

## Discussion

In this study, we utilized the LE-ESSI device equipped with FTICR MS to detect the distributions of DHA and DHA-containing lipid species on TBI tissue sections, along with high sensitivity and less background interference. It should be noted that a high nebulizing gas flowrate applied on the LE-ESSI device played an important role in reducing imaging hysteresis and that a combination of the LE-ESSI and FTICR MS is able to obtain high quality ion images, along with the good experimental reproducibility, reliable linearity, and high mass resolution. The lipids listed in Table S1 were confirmed based on the high mass accuracy, ultrahigh resolution, and/or *in situ* MS/MS analysis.

TBI is a multifaceted disease with long-lasting adverse neurological sequelae and diverse pathogenic mechanisms involved in numerous biomolecules^13^. Based on the cellular and molecular events induced by brain injury, TBI can be divided into acute and chronic phases^5–7^. Nowadays, the omega-3 fatty acids, such as DHA, which is typically recognized as a nutrient for brain, have been proven to perform neuro-restorative capacity for targeting the multiple phases^42–44^. Clinical study indicated that DHA supplement is helpful for brain injury recovery after TBI in a long term period^12^. DHA-related mechanisms remain unclear^13^, so *in situ* detection of the distribution of DHA on TBI brain tissue may provide some information for understanding secondary injury progression. Previous studies have observed increased ceramides and decreased sphingomyelins and PC(34:1) in the injured areas of TBI and injured ischemic brain using MALDI MSI, respectively^45, 46^. However, brain tissue-resident DHA has not been detected due to the interference of MALDI matrix and/or its clusters over the low mass range (typically <*m/z*500). In this study, the LE-ESSI equipped with FTICR MS has detected change trends of DHA and DHA-containing phospholipids in the injury location in TBI rats during the recovery process after TBI (Figs 4 and 5).These results suggest that the injured brain may urgently recruit DHA for repair and other related functions. In addition, changes in the size of the injury area based on the images of DHA indicate that the time points at 1 and 3 days after TBI are the acute phase of TBI (Supplementary Figure S6).

Previous study indicated that DHA plays important roles in decreasing inflammatory reaction and oxidative stress, neurotrophic support, and activation of cell survival pathways^14^. We found that increased DHA in the injury area may promote tissue protection and functional recovery at the acute phase after TBI (Fig. 4). These findings are consistent with the elevated plasma DHA levels at 1 day and decreased at 3 days after spinal cord injury^19, 47^, suggesting that the increased DHA in injured tissues might move into the cerebrospinal fluid and plasma. In addition, DHA-containing phospholipids including PE(P-18:0/22:6), PE(18:0/22:6), and PS(18:0/22:6), showed the opposite change trends in injury area between the acute and chronic stages. This finding is consistent with the decreased PE(40a:6) detected in injury area at 3 days after TBI and with decrease in the levels of arachidonic acid and DHA-containing lipid species in plasma from TBI model at the chronic phase^48^. Previous study found that lipases such as phospholipase A2 were activated under injury press to degrade membrane phospholipids, leading to the release of fatty acids such as DHA^14^, which is consistent with our detected increase of DHA in the injury areas after TBI. In addition, changes of DHA-containing lysophosphatides such as increased LPE(22:6) at 0, 1, and 3 days and decreased LPG(22:6) at 5 and 7 days in the injury areas after TBI are consistent with the increased level of lyso-phosphatidylcholine (16:0) in the area of focal cerebral ischemia^49^. Phospholipids can be hydrolyzed by phospholipase A2 to generate lysophosphatide, the DHA-containing LPE(22:6) and LPG(22:6) detected in our study may be the intermediate products of the degraded DHA-containing phospholipids. Our results suggest that alterations in the levels of DHA and DHA-containing lipid species in the injury areas closely correlated with the acute and chronic phases after TBI and the degree of injury.

## Conclusions

In this study, a combination of the LE-ESSI device and FTICR MS has exhibited a potential capability to image small molecules on tissue sections, along with high mass accuracy, ultrahigh mass resolution, high reproducibility, and a good linearity correlation between intensities and amounts of detected analytes. A series of ion images of DHA and DHA-containing lipid species on brain tissue sections from TBI model and normal control have revealed that DHA and DHA-containing lipid species play critical roles in the recovery process of TBI. More importantly, increased DHA in the injury area at the acute phase may be first required from the degraded DHA-containing lipid species for wound healing. Our results first provide the histological evidence of therapeutic potential of DHA in the treatment of TBI.

## Methods

### Chemicals and materials

PC(24:0) and PC(36:0) were purchased from Avanti Polar lipids (Alabaster, AL). HPLC-grade methanol and acetonitrile were supplied by Fisher Scientific (Pittsburgh, PA). Ultrapure water was purified using a Milli-Q system (Millipore, USA). Blue ink patterns printed on a commercial photo paper using a Canon ip 2780 inkjet printer (Tokyo, Japan) was used as a test sample.

### Traumatic Brain Injury Model

Adult male Sprague-Dawley rats (8-week-old, 225–300 g) were purchased from Shanghai Super-B&K laboratory animal Corp. Ltd (Shanghai, China) and kept in a 12/12 h light/dark cycle with access to food and water adlibitum. Animals were divided in two groups of normal control (n = 3) and injured group (n = 15).The injured group was further subdivided into five sub-groups, reflecting the time elapsed after trauma before the animals were sacrificed (at 0, 1, 3, 5, and 7 days). All groups including normal control, contained three rats for the repeated tests. The fluid percussion injury model in rats was employed as previously described^4, 45^. In detail, animals were anesthetized with 16% chloral hydrate (350 mg/kg) followed by fixing the skull on a stereotactic frame. Craniectomy was made over the left parietal cortex (3.0 mm posterior to bregma and 3.0 mm left of the midline) using a 3 mm diameter drill. Then the rats were subjected a fluid pressure strike (37 °C saline fluid) to the intact dura through the craniotomy at moderate severity (1.5–1.9 atmosphere, 13–15 ms duration) by using a fluid percussion device (Shanghai ALCBio, China). Subsequent to the injury, the bone flap was replaced and the incision was sutured, and the animals were allowed to recover for different time periods (0, 1, 3, 5, and 7 days) before they were anesthetized with isoflurane and decapitated. Control animals underwent the above-mentioned procedures of the craniotomy and anesthesia in the same duration without a fluid percussion strike. In addition, the chest cavity of each rat was opened quickly and 50 to 100 mL of phosphate buffered saline was perfused through ascending aorta at room temperature to flush blood from the head through an opening in the superior vena cava, and then the skull was carefully opened and brain tissue was taken out. The brains were carefully rinsed with saline water to remove blood outside, and then were snap-frozen and stored at −80 °C until sectioning. This study was approved by the Ethics Committee of Institute of Basic Medical Sciences, Chinese Academy of Medical Sciences. All experiments were performed in accordance with relevant guidelines and regulations.

### Tissue sample preparation

A series tissue sections (12 μm thickness) derived from 8-week-old CD1 mice (Vital River Laboratories, Beijing, China), TBI model and normal control were prepared at −20 °C using a cryostat (Leica CM3050, Leica Microsystems Inc., Wetzlar, Germany) followed by thaw-mounting on glass slides for the optimization of device conditions and MSI. The slides were stored in closed containers at −80 °C until MSI analysis. Adjacent sections were collected onto glass slides and performed with Nissl’s staining for comparisons of tissue morphology.

### LE-ESSI MSI

The LE-ESSI is composed of a liquid bridge and an ESSI source (Fig. 1A). The liquid bridge was constructed with one fused silica capillary (50 μm ID, 150 μm OD, SGE Analytical Science, Melbourne, Australia) used for solvent delivery (namely primary capillary) and another fused silica capillary for transferring extracted analytes into MS inlet (namely aspiration capillary), both of which were typically formed a 90 degree angle to build a stable liquid bridge between the two capillaries and sample surface. The ESSI source included the aspiration capillary tubing, an outer PEEK tube (360 μm ID, 1/16 in. OD, Upchurch Scientific, Oak Harbor), and a Swagelok PEEK tee. The aspiration capillary tubing was approximately 70 mm long, and the nebulizing gas was going through the gap between the aspiration capillary and the outer PEEK tube. The primary capillary was used to deliver solvent (60% methanol/30% acetonitrile/10% water, v/v/v) onto the sample surface, and a flowrate of 145 μL/h and a voltage of 2 kV were employed in extraction solvent. Sample on glass slide was positioned using a high-resolution XYZ stage (Kohzu Precision Co., Ltd, Kawasaki, Japan) controlled by a home-made interface. The liquid bridge and sample were monitored using a digital microscope (×500) during a whole experiment. All mass spectra were acquired using a 9.4 T Apex-ultra^TM^ hybrid Qh-FTICR MS (Bruker Daltonics, Billerica, MA) equipped with the LE-ESSI instead of a commercial ESI source. For MSI, the XY plane was moved continuously along the X-axis direction followed by being moved along the Y-axis direction with a fixed step. The scan rate at the X-axis direction was 100 μm/s at the fixed step of 150 μm for the Y-axis direction, along with the acquisition time of each spectrum of 1.5 s, a mass resolution of 200,000 (or 66,000) at *m/z* 400 and the *m/z* range of 600~1000 (or 200~1000) in positive (or negative) ion mode.

### Data processing and statistical analysis

Original MS data were continuously acquired using Apex Control 3.0.0 software (Bruker Daltonics, Billerica, MA) under a chromatography mode and the whole dataset was separated into individual mass spectrum corresponding to one pixel of image using the FTMS processing software (Bruker Daltonics, Billerica, MA). Ion images were reconstructed using the FlexImaging software (version 2.1, Bruker Daltonics) based on the absolute intensity of each ion and the injury area boundary was defined as 5-fold change in absolute intensity of the targeted ions. All analytes were identified based on their measured accurate masses with a mass error of <2 ppm relative to theoretical values and tandem mass spectra in combination with the available databases (the LIPID MAPS (http://www.lipimaps.org/) and the METLIN (http://metlin.scripps.edu/)) (Supplementary Table S1). For statistic analysis, peaks with signal-to-noise ratio, relative intensity, and absolute intensity thresholds of >2, 0.1%, and 10,000, respectively, were chosen as variables with reliable isotope distributions using Data Analysis 4.0 software (Bruker Daltonics). After isotopic deconvolution, peak intensities were normalized to 1000, and missing metabolites were imputed using the half of baseline value. GraphPad Prism software was used to draw box-chart and performed Wilcoxon-Mann-Whitney test. MetaboAnalyst tool (http://www.metaboanalyst.ca/) was used for orthogonal partial least squares discriminant analysis (OPLS-DA).

## Electronic supplementary material

Supplementary Information
