# Supplementary material for: Monitoring changes of docosahexaenoic acid-containing lipids during the recovery process of traumatic brain injury in rat using mass spectrometry imaging
Source: Sci Rep. 2017 Jul 11;7:5054. doi: 10.1038/s41598-017-05446-2 (PMC5506011; doi:10.1038/s41598-017-05446-2)
Supplement: Supplementary file 1 — Supplementary Information [file 41598_2017_5446_MOESM1_ESM.pdf]

Supplementary information:

Monitoring changes of docosahexaenoic acid-containing lipids during the recovery process of traumatic brain injury in rat using mass spectrometry imaging

Shuai Guo[1], Dan Zhou[1], Mo Zhang[1], Tiejun Li[2], Yujie Liu[1], Yupin Xu[1], Tianjing Chen[1] & Zhili Li[1],*

[1]Department of Biophysics and Structural Biology, Institute of Basic Medical Sciences, Chinese Academy of Medical Sciences & School of Basic Medicine, Peking Union Medical College, Beijing, PR China

[2]School of Pharmacy, Second Military Medical University, Shanghai, PR China

***Corresponding author: Zhili Li**, Department of Biophysics and Structural Biology, Institute of Basic Medical Sciences, Chinese Academy of Medical Sciences & School of Basic Medicine, Peking Union Medical College, 5 Dongdan San Tiao, Beijing 100005, PR China.

E-mail: lizhili@ibms.pumc.edu.cn,

Tel/Fax: +86-10-69156479.

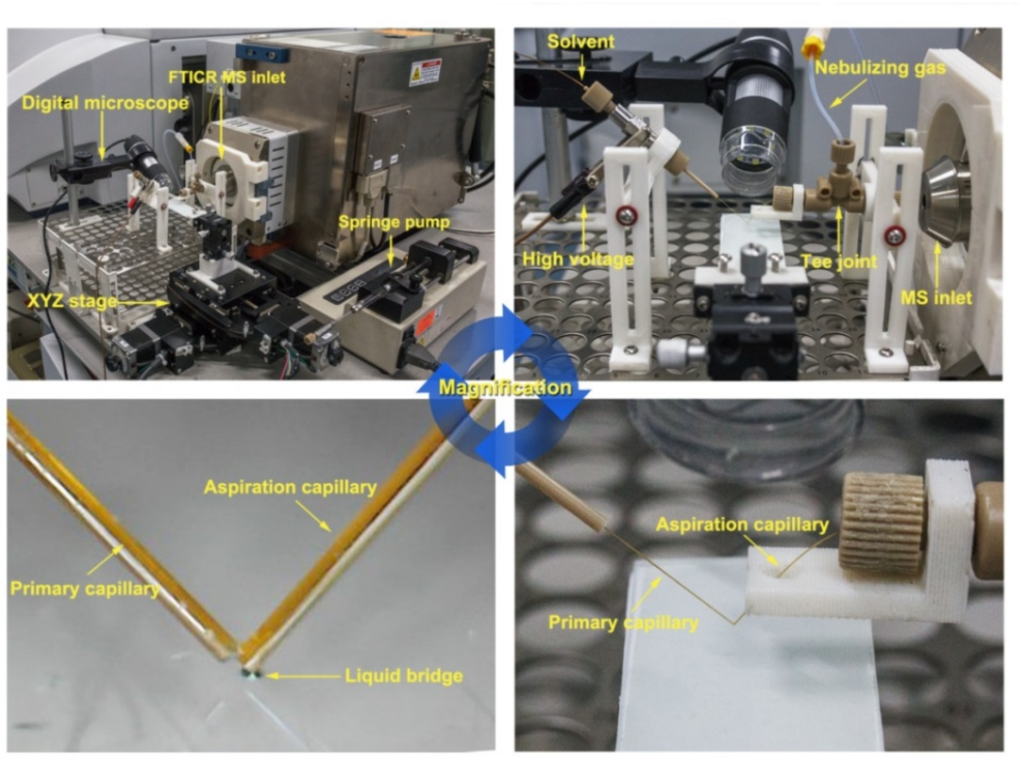

Figure S1. Physical photo of the LE-ESSI.

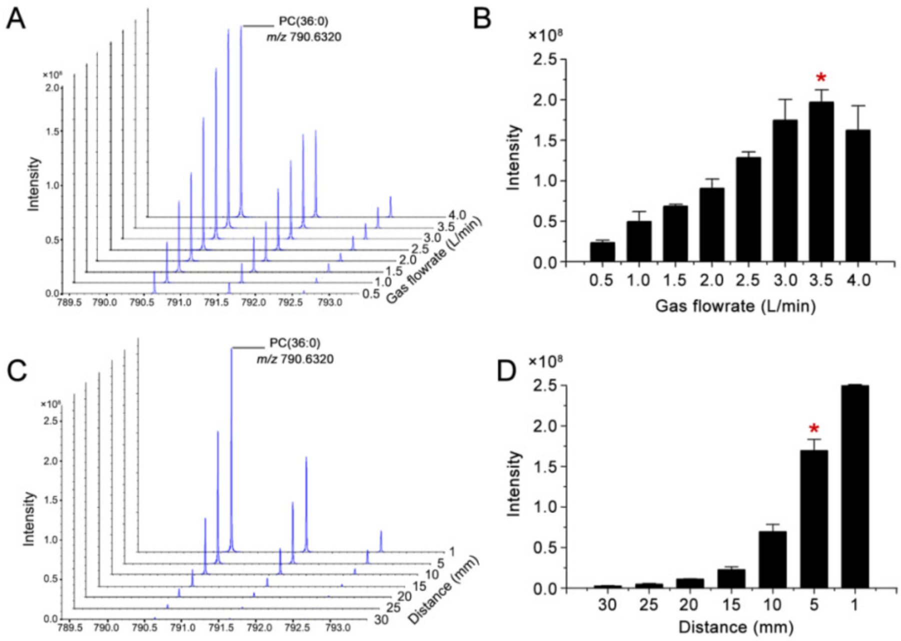

Figure S2. Optimizing nebulizing gas flowrate and the distance between spray tip and MS inlet using a PC(36:0) solution (3.2 fmol/µL) as a test sample. Mss spectra (A) and averaged signal intensities of PC(36:0) (B) at the different nebulizing gas flowrate. Mass spectra(C) and averaged signal intensities (D) at the different distances between pray tip and MS inlet. The liquid bridge was put into in a cup containing PC(36:0) solution. Error bars represent the standard deviation in triplicates in (B) and (D). The optimal conditions were marked with red asterisks.

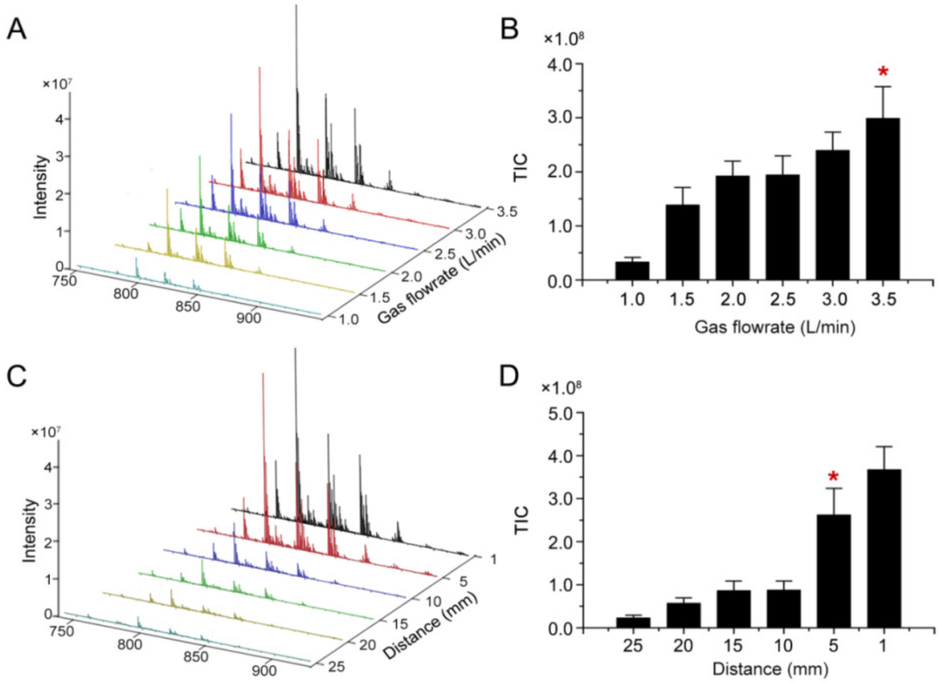

Figure S3. **Optimizing the nebulizing gas flowrate and the distance between spray tip and MS inlet using a homogeneous liver tissue as a test sample.** Mass spectra (A) and averaged total ion currents (TIC) (B) of liver tissue at different nebulizing gas flowrate. Mass spectra (C) and averaged TIC (D) versus the different distances between pray tip and MS inlet. The homogeneous mouse liver tissue was imaged by the LE-ESSI at a speed of 100 μm/s, and acquisition time of one spectrum was 1.5 s. Error bars represent the standard deviation of ~80 spectra in (B) and (D). The optimal conditions were marked with red asterisks.

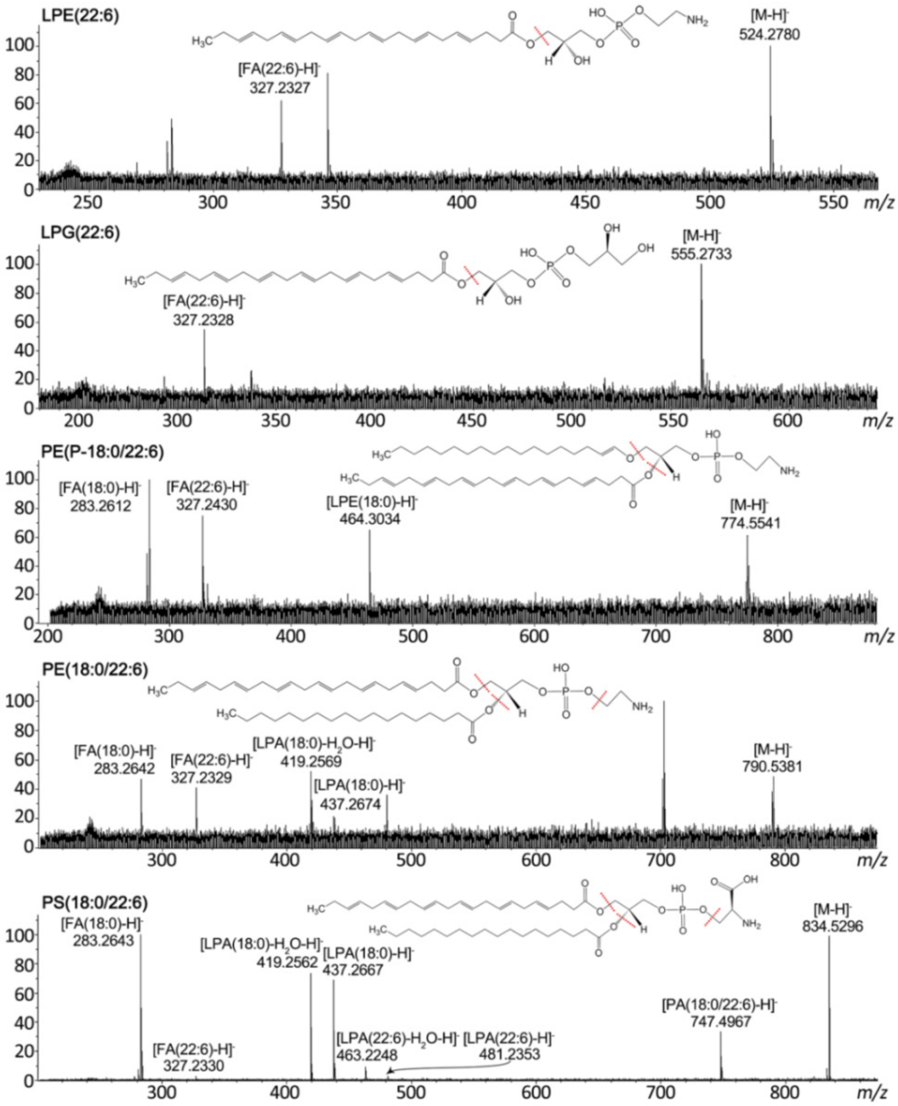

Figure S4. *In situ* MS/MS of LPG(22:6), LPE(22:6), PE(P-18:0/22:6), PE(18:0/22:6), and PS(18:0/22:6) on rat brain tissue sections.

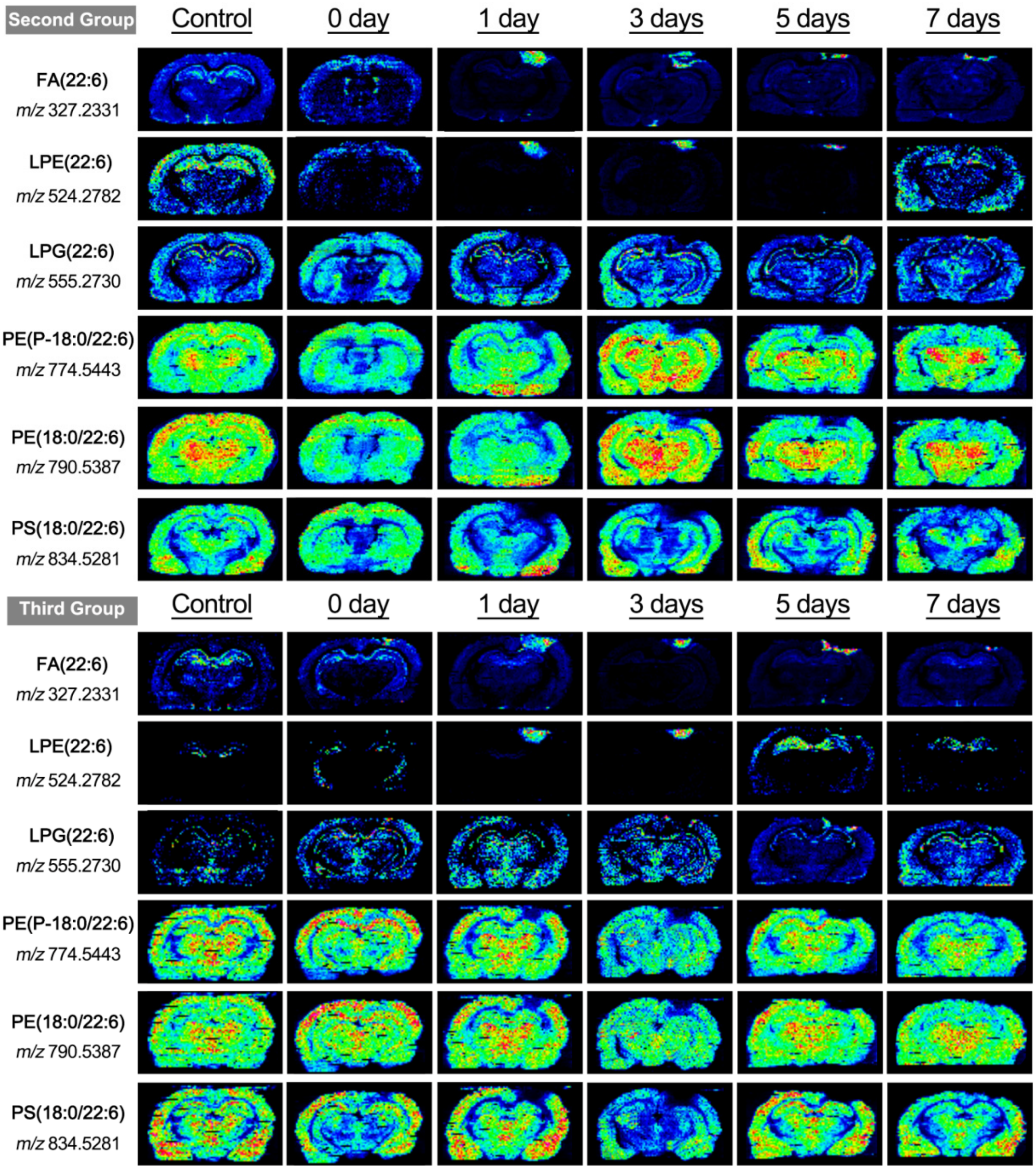

Figure S5. Ion images of the second and third TBI groups at 0, 1, 3, 5, and 7 days after injury.

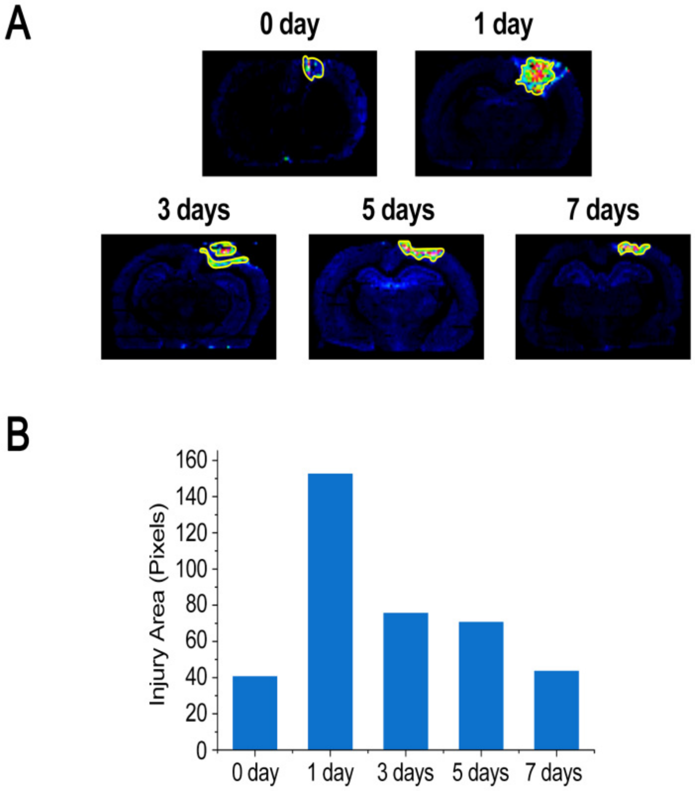

Figure S6. (A) Ion images of DHA at the different time points. (B) The image areas of DHA at the different time points. Area circled with yellow line represents the injury area.

Table S1. Identified analytes on rat brain sections by LE-ESSI-FTICR MS in negative ion mode.

Measured *m/z*	Calculated *m/z*	Error (ppm)	Assignment			MS/MS fragments *m/z*	
			Ion form	Compound	Molecular formula	Theoretical *m/z*	Sample *m/z*
253.2173	253.2173	0.00	$[M-H]^-$	FA(16:1)	C16H30O2		
255.2329	255.233	-0.39	$[M-H]^-$	FA(16:0)	C16H32O2		
267.0736	267.0735	0.37	$[M-H]^-$	Inosine	C10H12N4O5		
279.233	279.233	0.00	$[M-H]^-$	FA(18:2)	C18H32O2		
281.2486	281.2486	0.00	$[M-H]^-$	FA(18:1)	C18H34O2		
283.2643	283.2643	0.00	$[M-H]^-$	FA(18:0)	C18H36O2		
302.0663	302.0662	0.33	$[M+Cl]^-$	Inosine	C10H12N4O5		
303.233	303.233	0.00	$[M-H]^-$	FA(20:4)	C20H32O2		
308.0991	308.0987	1.30	$[M-H]^-$	Sialic acid	C11H19NO9		
309.2799	309.2799	0.00	$[M-H]^-$	FA(20:1)	C20H38O2		
317.2256	317.2253	0.95	$[M+Cl]^-$	FA(18:1)	C18H34O2		
327.2331	327.233	0.31	$[M-H]^-$	FA(22:6)	C22H32O2		
331.2644	331.2643	0.30	$[M-H]^-$	FA(22:4)	C22H36O2		
339.2098	339.2096	0.59	$[M+Cl]^-$	FA(20:4)	C20H32O2		
346.0559	346.0558	0.29	$[M-H]^-$	Adenosine monophosphate	C10H14N5O7P		
362.0508	362.0507	0.28	$[M-H]^-$	Guanosine monophosphate	C10H14N5O8P		
363.2097	363.2096	0.28	$[M+Cl]^-$	FA(22:6)	C22H32O2		
365.2464	365.2464	0.00	$[M+Cl]^-$	MG(16:0)	C19H38O4		
391.2622	391.2621	0.26	$[M+Cl]^-$	MG(18:1)	C21H40O4		
393.2565	393.2566	-0.25	$[M+Cl]^-$	FA(22:5)	C24H38O2		
413.2464	413.2464	0.00	$[M+Cl]^-$	MG(20:4)	C23H38O4		
435.252	435.2517	0.69	$[M-H]^-$	LPA(18:1)	C21H41O7P		
437.2465	437.2464	0.23	$[M+Cl]^-$	MG(22:6)	C25H38O4		
478.2937	478.2939	-0.42	$[M-H]^-$	LPE(18:1)	C25H38O4	478.2939/281.2486	478.2943/281.2487

500.2782	500.2783	-0.20	$[M-H]^-$	LPE(20:4)	C25H44NO7P	500.2783/303.233	500.2792/303.2327
509.2884	509.2885	-0.20	$[M-H]^-$	LPG(18:1)	C24H47O9P		
524.2782	524.2783	-0.19	$[M-H]^-$	LPE(22:6)	C27H44NO7P	524.2783/327.233	524.2777/327.233
531.2725	531.2728	-0.56	$[M-H]^-$	LPG(20:4)	C26H45O9P	531.2728/303.233	531.2742/303.233
544.2673	544.2681	-1.47	$[M-H]^-$	LPS(20:4)	C26H44NO9P		
555.273	555.2728	0.36	$[M-H]^-$	LPG(22:6)	C28H45O9P	555.2728/327.233	555.2733/327.2328
558.0642	558.0644	-0.36	$[M-H]^-$	Adenosine diphosphate ribose	C15H23N5O14P2		
559.4727	559.4726	0.18	$[M-H20-H]^-$	DG(33:2)	C36H66O5		
568.2681	568.2681	0.00	$[M-H]^-$	LPS(22:6)	C28H44NO9P		
572.4812	572.4815	-0.52	$[M+Cl]^-$	Cer(d34:1)	C34H67NO3		
585.4886	585.4883	0.51	$[M-H20-H]^-$	DG(35:3)	C38H68O5		
598.4971	598.4971	0.00	$[M+Cl]^-$	Cer(d36:2)	C36H69NO3		
599.3202	599.3202	0.00	$[M-H]^-$	LPI(18:0)	C27H53O12P		
619.2887	619.2889	-0.32	$[M-H]^-$	LPI(20:4)	C29H49O12P	619.2889/303.233	619.2886/303.2329
627.4758	627.4761	-0.48	$[M+Cl]^-$	DG(34:2)	C37H68O5		
631.4704	631.4708	-0.63	$[M-H]^-$	LPA(32:1)	C35H68O7P		
673.4814	673.4814	0.00	$[M-H]^-$	PA(34:1)	C37H71O8P		
679.508	679.5074	0.88	$[M+Cl]^-$	DG(38:4)	C41H72O5		
699.4972	699.497	0.29	$[M-H]^-$	PA(18:1/18:1)	C39H73O8P	699.497/281.2486	699.4934/281.2485
700.5287	700.5286	0.14	$[M-H]^-$	PE(P-34:1)	C39H76NO7P		
701.5125	701.5127	-0.29	$[M-H]^-$	PA(18:1/18:0)	C39H75O8P	701.5127/437.2663/419.2557 /283.2643/281.2486	701.5128/437.2657/419.2562 /283.2643/281.2486/
705.5069	705.5076	-0.99	$[M-H]^-$	LPG(32:1)	C38H74O9P		
709.481	709.4814	-0.56	$[M-H]^-$	PA(37:4)	C40H71O8P		
716.5233	716.5236	-0.42	$[M-H]^-$	PE(34:1)	C39H76NO8P		
719.4653	719.4657	-0.56	$[M-H]^-$	PA(38:6)	C41H69O8P		
721.5049	721.5057	-1.11	$[M+Cl]^-$	PE-Cer(36:2)	C38H75O10P		
722.5133	722.513	0.42	$[M-H]^-$	PE(P-36:4)	C41H74NO7P		
726.5438	726.5443	-0.69	$[M-H]^-$	PE(P-36:2)	C41H78NO7P		
728.5605	728.5599	0.82	$[M-H]^-$	PE(P-36:1)	C41H80NO7P		

733.4814	733.4814	0.00	$[M-H]^-$	PA(39:6)	C42H71O8P		
735.4732	735.4737	-0.68	$[M+Cl]^-$	PA(36:2)	C39H73O8P		
742.5398	742.5392	0.81	$[M-H]^-$	PE(18:1/18:1)	C41H78NO8P	742.5392/281.2486	742.5394/281.2484
744.5543	744.5549	-0.81	$[M-H]^-$	PE(36:1)	C41H80NO8P		
746.5129	746.513	-0.13	$[M-H]^-$	PE(P-38:6)	C43H74NO7P		
747.5192	747.5181	1.47	$[M-H]^-$	PG(18:0/16:1)	C40H77O10P	747.5181/437.2663/419.2557/283.2643	747.5182/437.2659/419.2561/283.2643
749.5334	749.5338	-0.53	$[M-H]^-$	PG(34:0)	C40H79O10P		
750.5444	750.5443	0.13	$[M-H]^-$	PE(P-18:0/20:4)	C43H78NO7P	750.5443/464.3146/303.2330/283.2643	750.5450/464.3157/303.2332/283.2641
754.576	754.5756	0.53	$[M-H]^-$	PE(P-38:2)	C43H82NO7P		
760.5126	760.5134	-1.05	$[M-H]^-$	PS(34:1)	C40H76NO10P		
762.5074	762.5079	-0.66	$[M-H]^-$	PE(38:6)	C43H74NO8P		
766.5401	766.5392	1.17	$[M-H]^-$	PE(18:0/20:4)	C43H78NO8P	766.5392/718.5392/480.3095/303.2330/283.2643	766.5403/718.5385/480.3086/303.2329/283.2642
769.5025	769.5025	0.00	$[M-H]^-$	PG(36:4)	C42H75O10P	769.5025/303.2330/255.2330	769.5021/303.2329/255.2333
770.4985	770.4977	1.04	$[M-H]^-$	PS(35:3)	C41H74NO10P		
774.5439	774.5443	-0.52	$[M-H]^-$	PE(P-18:0/22:6)	C45H78NO7P	774.5443/464.3146/327.2330/283.2643	774.5048/464.3144/327.2328/283.2431
777.5648	777.5651	-0.39	$[M-H]^-$	PG(36:0)	C42H83O10P		
778.5758	778.5756	0.26	$[M-H]^-$	PE(P-18:0/22:4)	C45H82NO7P	778.5756/331.2643	788.5758/331.2643
786.5287	786.529	-0.38	$[M-H]^-$	PS(18:1/18:1)	C42H78NO10P	786.5290/699.4970/435.2517/417.2406/281.2486	786.5283/699.4959/435.2517/417.2411/281.2487
788.5442	788.5447	-0.63	$[M-H]^-$	PS(18:0/18:1)	C42H80NO10P	788.5447/701.5127/437.2663/419.2557/283.2643/281.2486	788.5441/701.5126/437.2675/419.2567/283.2640/281.2484
790.5387	790.5392	-0.63	$[M-H]^-$	PE(18:0/22:6)	C45H78NO8P	790.5392/701.5127/437.2663/419.2557/327.2330/283.2643	790.5381/701.5129/437.2674/419.2569/327.2329/283.2642
794.5472	794.5472	0.00	[M+Cl]-	PC(16:0/18:1)	C42H82NO8P	794.5472/744.5549/281.2486/255.2330	794.5476/744.5545/281.2485/255.2329
797.5334	797.5338	-0.50	$[M-H]^-$	PG(18:0/20:4)	C44H79O10P	797.5338/303.2330	797.5324/303.2329
808.512	808.5134	-1.73	$[M-H]^-$	PS(38:5)	C44H76NO10P		
810.5281	810.529	-1.11	$[M-H]^-$	PS(18:0/20:4)	C44H78NO10P	810.5290/723.4970/437.2663	810.5271/723.4969/437.2678

						/419.2557/283.2643	/419.2572/283.2646
814.5605	814.5603	0.25	$[M-H]^-$	PS(38:2)	C44H82NO10P		
818.5351	818.5341	1.22	$[M-H]^-$	PS(P-40:6)	C46H78NO9P		
819.5177	819.5182	-0.61	$[M-H]^-$	PG(40:7)	C46H77O10P		
821.5463	821.5469	-0.73	[M+Cl]-	PG(P-38:2)	C44H83O9P		
822.5788	822.5785	0.36	[M+Cl]-	PC(18:1/18:0)	C44H86NO8P	822.5785/772.5862/281.2486	822.5788/772.5852/281.2486
834.5281	834.529	-1.08	$[M-H]^-$	PS(18:0/22:6)	C46H78NO10P	834.5290/747.4970/481.2361 /463.2250/437.2663/419.2557 /327.2330/283.2643	834.5275/747.4964/481.2363 /463.2254/437.2674/419.2567 /327.2329/283.2641
838.5607	838.5603	0.48	$[M-H]^-$	PS(18:0/22:4)	C46H82NO10P	838.5603/751.5283/437.2663 /419.2557/331.2643/283.2643	838.5604/751.5290/437.2672 /419.2567/331.2640/283.2642
857.5169	857.5185	-1.87	$[M-H]^-$	PI(16:0/20:4)	C45H79O13P	857.5185/553.2778/391.2255 /303.2330/255.2330	857.5170/553.2771/391.2259 /303.2324/255.2330
865.5015	865.5025	-1.16	$[M-H]^-$	PG(44:12)	C50H75O10P		
878.4989	878.4977	1.37	$[M-H]^-$	PS(44:12)	C50H74NO10P		
881.528	881.5186	0.57	$[M-H]^-$	PI(38:6)	C47H79O13P		
882.5291	882.529	0.11	$[M-H]^-$	PS(44:10)	C50H78NO10P		
883.5353	883.5342	1.24	$[M-H]^-$	PI(18:1/20:5)	C47H81O13P	883.5342/579.2934/417.2406 /303.2330/281.2486	883.5352/579.2930/417.2414 /303.2332/281.2482
885.55	885.5498	0.23	$[M-H]^-$	PI(18:0/20:4)	C47H83O13P	885.5498/599.3202/581.3091 /439.2250/419.2557/303.2330 /283.2643	885.5514/599.3205/581.3094 /439.2246/419.2566/303.2329 /283.2641
909.548	909.5498	-1.98	$[M-H]^-$	PI(40:6)	C49H82O13P		
939.536	939.5369	-0.96	$[M-H]^-$	PIP(P-37:2)	C46H85O15P		

Some metabolites were not confirmed by MS/MS.

Table S2. Comparison of DHA and DHA-containing lipid species in normal control and TBI rats at 0, 1, 3, 5, and 7 days after the injury.

Lipids	Kruskal-Wallis test															
	Summary	NC *vs.*D0	NC *vs.*D1	NC *vs.*D3	NC *vs.*D5	NC *vs.*D7	D0 *vs.*D1	D0 *vs.*D3	D0 *vs.*D5	D0 *vs.*D7	D1 *vs.*D3	D1 *vs.*D5	D1 *vs.*D7	D3 *vs.*D5	D3 *vs.*D7	D5 *vs.*D7
FA(22:6)	***	*	***	***	***	***	***	***	***	ns	***	***	***	ns	ns	*
LPE(22:6)	***	***	***	***	ns	ns	ns	ns	***	***	ns	***	***	***	***	ns
LPG(22:6)	***	ns	***	*	***	***	***	ns	***	***	***	***	***	***	***	ns
PE(P-18:0/22:6)	***	ns	***	***	***	***	***	***	***	ns	*	ns	***	ns	ns	***
PE(18:0/22:6)	***	ns	***	***	***	***	***	***	***	***	**	ns	*	ns	ns	ns
PS(18:0/22:6)	***	***	***	***	***	***	***	**	ns	ns	**	***	***	ns	*	ns

NC: normal control.

D0, D1, D2, D3, D5, and D7 represent 0, 1, 2, 3, 5, and 7 days, respectively.

*, $p < 0.05$; **, $p < 0.01$; ***, $p < 0.001$.

ns: no significant difference.
